# Do Parental Expectations Play a Role in Children's Sleep and Mothers' Distress? An Exploration of the Goodness of Fit Concept in 54 Mother-Child Dyads

**DOI:** 10.1155/2011/104832

**Published:** 2012-01-11

**Authors:** Helene Werner, Oskar G. Jenni

**Affiliations:** ^1^Child Development Center, University Children's Hospital Zurich, Steinwiesstrasse 75, 8032 Zurich, Switzerland; ^2^Children's Research Center, University Children's Hospital Zurich, Steinwiesstrasse 75, 8032 Zurich, Switzerland

## Abstract

This study describes parental expectations for sleep-wake patterns in healthy kindergarten children and explores their relation to children's sleep quality and parental distress. Data analysis of 54 mother-child dyads (age range of the children: 4–7 years) indicated that parental expectations for children's sleep-wake patterns differ between scheduled and free days and depend on children's chronotype. Mothers of children with late chronotype showed less adequate expectations for children's sleep onset time than mothers of children with early chronotype (e.g., morning types). Furthermore, children of mothers with less adequate expectations for children's sleep onset time on scheduled days had longer settling periods during which sleep rituals may take place (*r* = 0.31, *P* ≤ 0.05), spent more time in bed than they actually sleep (*r* = 0.35, *P* ≤ 0.01), and had more frequently difficulties falling asleep (*r* = 0.33, *P* ≤ 0.01). However, less adequate expectations for children's sleep onset time were not associated with parental distress (*P* > 0.05). We conclude that parental expectations about their children's sleep play a key role in understanding normal and abnormal sleep during childhood.

## 1. Introduction

Children's sleep is embedded in the context of the family with its characteristics of size, lifestyle, parental personality, and parenting styles. Thus, the sleep behavior of the child results from a complex interplay between culture and biology [1, 2]. A detailed overview of involved components, dynamic interactions, and bidirectional influences between the child and the social environment is illustrated in the transactional model for children's sleep of Sadeh and Anders [3].

Children become skilled in sleep practices and learn specific sleep routines (e.g., cosleeping, bedtime rituals) as the normal and adequate ones. While some parents shape their parenting strategies and sleep routines in response to the child's biology and socioemotional needs, others primarily follow the accepted cultural standards (e.g., bringing the child to bed at 8 p.m. or letting the child to fall asleep alone). Following Thomas and Chess' principle [4, 5] that individuals must match their personal characteristics with the demands of their context and that positive feedbacks and normal development occur, when the child's characteristics are in accordance with environmental expectations and demands (goodness of fit), we investigated the relationship between parental expectations for children's sleep-wake patterns, children's sleep quality, and parental distress in a normative sample of healthy kindergarten children.

Sleep problems and parental complaints about difficulties falling asleep or frequent night wakings are very common during childhood [6]. However, the boundaries between “normal” and “problematic” sleep are often defined and shaped by cultural norms, expectations, and values. In fact, while some children can adapt and cope with certain standards well, a significant number of children struggle. Thus, at least some parent-reported sleep problems during childhood are created by specific cultural practices, which may be incongruent with aspects of individual sleep biology or with stages of children's socioemotional development [1, 2]. Although evidence is accumulating for a relationship between children's sleep and parental cognition [7, 8], the role of parental expectations acting as social time cues (zeitgebers) on individual's sleep is underestimated.

Following our clinical experience in the care of sleep-disordered children, we hypothesized that parents of a child with late chronotype would have less adequate expectations for their children's bed time and that these children may have poorer sleep quality and spend more time in bed than they actually sleep (because of too early bedtimes). Furthermore, we assumed that situations in which the child shows resistance to sleep or does not fall asleep as it is expected might be stressful for the parents. In line with this, we hypothesized that less adequate expectations for children's sleep-wake patterns are associated with higher parental distress.

The purpose of this study was (1) to describe parental expectations for children's sleep-wake patterns in a normative sample of healthy kindergarten children, (2) to examine whether parental expectations for children's sleep-wake patterns differ between scheduled and free days and (3) depend upon children's chronotype, and finally (4) to explore whether they are associated with children's sleep quality and mothers' distress.

## 2. Methods

### 2.1. Subjects

Overall, 660 families from 34 kindergarten schools in the greater Zurich area were invited by a study letter to participate in the study. Of the 68 families which finally agreed to participate, 54 children [24 girls and 30 boys, mean age 5.9 ± 0.6 (SD) years, range 4–7 years] and their primary caregivers [age 38.7 ± 4.2 (SD) years, range 31–48 years] were included in the analysis. Data were collected for the mothers of the child; these were for 43 of the 54 families (80%) the primary caregiver of the child (the person who spends most of the time with the child) while for 11 (20%) participants mothers and fathers were equally involved. Overall, 5 families were dropped from the study due to insufficient language skills, and 9 families were excluded because of (a) missing data or deregistration [*n* = 6; father was single parent and served as informant (*n* = 1); mother had night-shift work schedules (*n* = 1); no biological mother data (*n* = 1); missing parental distress data (*n* = 1); deregistration (*n* = 2)] and (b) mothers had two kindergarten children of which both children took part in the study, but only one child was included in the analysis based upon random selection (*n* = 3). At time of assessment, 47 of 54 mothers (83%) were married and lived with the child's father, 5 (9%) were single parent, and 2 (4%) were divorced and lived with a new partner. Informants reported neither organic or severe behavioral sleep disorders nor any sleep medication. Further sample characteristics are presented in Table 1.

All participating families received a letter including a description of the study and a study enrolment form. The study procedure was explained by the researchers, and written informed consent was obtained from all parents. The interview was conducted by a trained psychologist (HW) in the parents' home; questionnaires were filled out prior to the interview. Families participating in the study were rewarded with a gift certificate from a book shop. The study was approved by the Institutional Review Board (IRB) of the Canton Zurich and was performed according to the Declaration of Helsinki.

### 2.2. Measures

#### 2.2.1. Sleep-Wake Patterns and Chronotype

Children's *actual* sleep-wake patterns and circadian preference (chronotype) were assessed by the Children's ChronoType Questionnaire (CCTQ; [9]; German version). Sleep-wake patterns were assessed separately for scheduled days (SC; defined as those days when individual's sleep-wake patterns are directly influenced by individual's activities) and free days (FR; defined as days “free” from any influence of social time cues as, e.g., school). Computed variables included: (a) *sleep onset* defined as sleep latency added to time of lights off; (b) *sleep period* defined as the difference between sleep onset and get-up time; (c)* time in bed* defined as the difference between bed time and get up time; (d) *settling period* defined as the difference between sleep onset time and bed time; (e) *mid sleep point* (MSP) defined as sleep onset + sleep period/2. Children's chronotype is estimated by the MSP on FR (MSF) and by a CT score. The CT score is a single-item measurement assessing children's chronotype on a 5-point Likert scale [definitely a morning type (1), rather a morning type than an evening type (2), neither nor (3), rather an evening type than a morning type (4), definitely an evening type (5)]. The parents rated the chronotype based on a short text about sleep- and wake-related behaviors. Because many individuals compensate for a sleep deficit accumulated during SC by sleeping in on FR, MSF was corrected for the confounding sleep deficit based on the individual weekly average sleep period (MSFs; for correction algorithm, see supplement to [10]).

#### 2.2.2. Sleep Quality

Children's and mothers' overall sleep quality was rated by the mothers on a 10-point Likert scale ranging from very bad (1) to very good (10). A score <6 is considered as poor sleep quality and a score ≥6 as good sleep quality [11]. Children's *time in bed without sleeping* was defined by differences between time in bed and sleep period. Parents were also asked about behavioral sleep problems of their children. On one side the parents were asked whether the child has difficulties falling asleep and on the other hand whether the child frequently wakes up at night. Responses were rated by the interviewer as “no/rarely” if problems occurred less than twice a week, “sometimes” for 3 to 4 times per week, and “often” for 5 to 7 times per week.

#### 2.2.3. Parental Expectations

Parental expectations for children's sleep-wake patterns were assessed by asking the mothers during an interview about the children's *expected* (i.e., desired) sleep-wake patterns: what would be, in their opinion, the *expected* time for the child to go to bed, put off lights, and get up as well as for sleep latency. Except for sleep latency, the *expected* sleep-wake patterns were also questioned separately for SC and FR. Computed *expected* variables included sleep onset, sleep period, time in bed, settling time, and MSP and were identical defined as in the CCTQ. Larger differences between *actual* (assessed by the CCTQ) and *expected* sleep times would indicate less adequate parental expectations and could be a source of parenting distress (we call this difference the expectation discrepancy).

#### 2.2.4. Parental Distress

Mothers' parenting distress was assessed by the Parenting Stress Index-Short Form (PSI-SF), a 36-item self-report measure [12]. The PSI-SF includes a sum scale *Total Stress* (TS) and 3 subscales reflecting parents' perception of (1) child-rearing competence in relation to personal factors (*Parent Distress*, PD), (2) (dis)satisfaction with their interaction with the child (*Parent-Child Dysfunctional Interaction*, P-CDI), and (3) child's temperament, demandingness, and noncompliance (*Difficult Child*, DC). The PSI-SF has been shown to be a reliable and valid indicator of parenting distress [13, 14].

#### 2.2.5. Temperament

Children's temperament is an important covariate in relation to parental distress. For this reason we adjusted parental distress as a dependent variable by the measures of temperament. Children's temperament was assessed by the Emotionality-Activity-Sociability temperament inventory (EAS) [15], a 20-item parent-report measure for 1- to 9-year-old children measuring 4 temperament dimensions: (1) *Emotionality*: tendency to become aroused easily and intensely, (2) *Shyness*: tendency to be inhibited and awkward in new social situations, (3) *Sociability*: tendency to prefer the presence of others than to being alone, and (4) *Activity*: preferred levels of activity and speed of action. The EAS is proved to be a reliable instrument, with satisfying internal consistencies and good interrater agreement [16, 17].

#### 2.2.6. Socioeconomic Status

Paternal occupation and maternal education were gathered from mothers applying a 6-point Likert scale. Summing up these scores, a socioeconomic status (SES) ranging from 2 to 12 was obtained. Based on their SES score, children's families were allocated to one of the following three classes: lower class (scores 2 to 5), middle class (scores 6 to 9), and upper class (scores 10 to 12). This measure has been used in previous studies and has been proved to be a reliable and valid indicator of SES in Switzerland [18–20].

#### 2.2.7. Life Events

Similarly as for children's temperament, life events are an important covariate in relation to parental distress and for which we adjusted. Occurrence of 17 life events about changes in the work, financial, and private situations [21, 22] experienced by the family during the 12 months prior to the interview was assessed by mothers' report. A sum score was calculated representing the numbers of the family's life events (0–17). In addition, a total impact score was calculated. The impact was rated on a 5-point Likert scale ranging from 1 (mild) to 5 (severe) for each item.

### 2.3. Statistical Analysis

Descriptive results are presented as means and standard deviations (SD). Because most CCTQ variables showed significant skewness and/or kurtosis, we used nonparametric tests for all parameters for testing equality of means (Wilcoxon-Test) and Spearman correlations to measure associations between *actual* sleep-wake patterns, parental expectations for children's sleep-wake patterns, chronotype, sleep quality parameters, and parental distress scores. Kendall correlations were used to measure associations if one of the variables was dichotomized. Mean differences between SC and FR and between *actual* and *expected* sleep-wake patterns were analyzed by two-way analysis of variance for repeated measures. Linear regression models were used to describe the relationship between sleep-wake patterns, parental expectations for children's sleep-wake patterns, chronotype, sleep quality, and parental distress scores. The independent variables of the multiple regression analyses were chosen on the basis of significant univariate correlations. All analyses were performed with two-tailed tests, and *P* < 0.05 was considered significant. SPSS (19.0J for Windows; SPSS Inc., Chicago, IL, USA) was used for all statistical analyses.

## 3. Results

### 3.1. Parental Expectations for Children's Sleep-Wake Patterns

Parental expectation for children's sleep-wake patterns was estimated by *expected *sleep-wake patterns and the adequacy of the parental expectations by the differences between *actual* and *expected* sleep times (*actual-expected*). Larger differences between *actual* and *expected* sleep times would indicate less adequate expectations (also called expectation discrepancy). Differences between *actual* and *expected* sleep-wake patterns as well as between SC and FR were analyzed by 2-way analysis of variance for repeated measures (Table 2). For bed time, time of lights off, sleep onset, get-up time, and MSP, there was a significant interaction between the 2 factors (*actual/expected* and SC/FR) indicating that differences between *actual* and *expected* sleep-wake patterns were not equal for SC and FR. On the other hand, for sleep period, time in bed, and settling period there was no signficant interaction between the 2 factors. This finding means that the difference between *actual* and *expected* was the same for SC and FR, or, similarly, the difference between SC and FR was the same for *actual* and *expected*. In fact, for bed time (as well as for time of lights off, sleep onset time, get up time and MSP), mothers' *actual* and *expected* bedtime are nearly identical on FR whereas on SC mothers expect their children to go to bed earlier than they actually do. For sleep period the results indicate that children sleep less than mothers expect, but both *actual* and *expected* sleep periods increased from SC to FR.

Parental expectation discrepancy for children's bedtime, time of lights off, sleep onset, and get-up time (SC + FR) were significantly related to children's chronotype as measured by CT scores, with larger differences between *actual* and *expected* for later chronotypes. After including children's age, gender, and SES in the regression analysis, the effects remained significant. For better understanding, we present, together with the correlation between the CT scores and parental expectation discrepancy, the averages of sleep onset time and get-up time in the groups of “morning”, “neither nor”, and “evening” types in Table 3.

### 3.2. Parental Expectations for Children's Sleep-Wake Patterns in relation to Children's Sleep Quality Parameters

Sleep problems as difficulties falling asleep or frequent night wakings are common during childhood [6]. The children included in our sample did not experience severe behavioral sleep problems, although some children exhibited difficulties falling asleep or frequently woke up at night (Table 4).

We hypothesized that parental expectations for children's sleep onset time may influence children's sleep in the sense that less adequate expectations (larger differences between *actual* and *expected*) would be associated with poorer sleep quality of the children. In our sample, parental expectation discrepancy for children's sleep onset time (SC and FR) was significantly related to children's settling period and time in bed without sleeping (for statistics see Table 5). Furthermore, a longer settling period and longer time in bed without sleeping were also associated with later *actual* sleep onset time (e.g., for settling period on SC: *r* = 0.61, *P* ≤ 0.001; for time in bed without sleeping on SC: *r* = 0.55, *P* ≤ 0.001). Including *actual* and *expectation discrepancy* for sleep onset time in a linear regression analysis, multiple correlation indicated that both variables independently contribute to children's settling period (for SC: multiple correlation *R* = 0.73, *P* ≤ 0.001, for FR: *R* = 0.63, *P* ≤ 0.001) and to time in bed without sleeping (for SC: multiple correlation *R* = 0.71, *P* ≤ 0.001, for FR: *R* = 0.62, *P* ≤ 0.001). These effects remained significant after controlling for children's chronotype.

 For the other sleep quality parameters the results are not clear-cut and differed between SC and FR (Table 5). Parental expectation discrepancy for children's sleep onset time on FR was significantly associated with children's overall sleep quality assessed on a 10-point Likert scale while no significant association was found for SC. Poorer parent-reported overall sleep quality was related to less adequate expectations for sleep onset times on FR. Furthermore, Kendall correlation indicated that the frequency of difficulties falling asleep was significantly related to parental expectation discrepancy for sleep onset time on SC. Children of mothers with less adequate expectations for children's sleep onset time had more frequent difficulties falling asleep than children of mothers with more adequate expectations. No significant association was found between parental expectation discrepancy for sleep onset time and night waking (*P* > 0.05).

### 3.3. Association between Parental Expectations for Children's Sleep-Wake Patterns and Parental Distress

In line with Thomas and Chess [4, 5], we hypothesized that less adequate parental expectations for children's sleep-wake patterns may be associated with difficult parent-child interactions (subscale P-CDI) and higher parental distress (TS; for descriptive statistics see Table 1). Parental distress may be attributed to unique aspects of the child-rearing context. In our sample, numbers and total impact by life events, family size, mothers' age and employment, marital status, and SES were not related to parental distress. Moreover, children's age was not associated with parental distress, while children's sex, temperament, and mothers' sleep quality were. Including these 3 variables in a regression analysis, a significant effect of emotionality (*B* = 0.56, *P* ≤ 0.001) and mothers sleep quality (*B* = −0.43, *P* ≤ 0.001), but no significant sex effect (*B* = −0.18, *P* = 0.07) on parental distress (TS) were found. Higher emotionality of the children and poorer maternal sleep quality were associated with higher parental distress.

Parental expectations for children's sleep-wake patterns were not univariately associated with parental distress (TS and subscales: *P* > 0.05). However, on the basis of the previous results, *actual* sleep-wake patterns (bed time, time of lights off, sleep onset), expectation discrepancy for these three sleep-wake patterns as well as children's chronotype, emotionality, and mothers' sleep quality were included in a multiple regression analyses (Table 6). After controlling for these variables, parental expectation discrepancy for any of the three sleep-wake patterns was not significantly associated with parental distress (TS and subscales: *P* > 0.05; in Table 6 only the effects for the subscale P-CDI is presented). No effects were also found for free days (FR).

## 4. Discussion

On the basis of “goodness of fit” concept [4, 5], we investigated parental expectations for children's sleep-wake patterns in a normative sample of healthy kindergarten children and explored their relation to children's sleep quality and parental distress as a proxy for children's general developmental opportunities and psychosocial adjustment. Although evidence is accumulating for a link between children's sleep and parental cognition [7, 8], the influence of parental expectations which may act as social time cues (zeitgebers) on children's sleep is still largely underestimated. Following our clinical experience in the care of sleep-disordered children, we defined parental expectations as *expected* sleep-wake patterns and assumed that large differences between *actual* and *expected* sleep-wake behavior indicate less adequate expectations and could be a source of parenting stress.

Our findings show that parental expectations differ between scheduled and free days for many sleep-wake patterns of the children. For bed time (and also for time of lights off and sleep onset time), expected and actual times were nearly identical on FR, whereas on SC they differed. On one side mothers expected their children to go to bed earlier on SC and on the other hand to get-up later on FR than they actually do. In our sample, less adequate expectations for children's sleep onset time and get up time were significantly associated with children's chronotype. Mothers of children with late chronotypes (so-called evening types or “owls”) have more frequently less adequate expectations for sleep onset time than mothers of children with early chronotypes (so-called morning types or “larks”). Individuals' chronotype contributes considerably to differences in habitual sleep rhythms. “Morning people” show a preference for waking at an early hour and find it difficult to remain awake beyond their usual bedtime, as compared with “night people”, who show a preference for sleeping at later hours and often find it difficult to get up in the morning. We believe that social institutional demands, such as standardized school times, substantially influence parental expectations for children's sleep-wake patterns. Mothers of children with late chronotype may experience higher pressure to bring their children to bed on time—in particular during SC (i.e., school days)—than mothers of children with early chronotype.

In contrast to our hypothesis, less adequate parental expectations were not significantly associated with higher parental distress. Mothers' distress was measured by the PSI-SF, a valid and widely used questionnaire to identify parent-child systems under stress and at risk for dysfunctional parenting [12]. Although the full-length PSI examines the parent-child dyad more closely, the short form appears to capture the primary components of parenting distress. However, parents' level of distress is influenced by a wealth of factors (e.g., personality, social support, marital satisfaction). In our study, mothers' distress was not related to their age, family size, frequency of life events, and SES, while mothers of children with higher emotionality and mothers with poorer sleep quality had higher distress. Including children's emotionality, chronotype, and actual sleep-wake patterns as well as mothers' sleep quality and parental expectation discrepancy for children's sleep-wake patterns in multiple regression analyses, no significant association of parental expectation discrepancy with parental distress was found. Thus, children's emotionality and mother's sleep quality contribute stronger to the level of parenting distress than parental expectations or actual sleep-wake patterns. We suppose, however, that no significant association between parental expectation discrepancy and distress was found due to the limited power of our sample size with non-sleep-disordered children (parents of sleep-disordered children may experience higher distress). Future studies should be performed on clinical samples, should also incorporate additional determinants of parental distress (e.g., social support by spouse, family members, or friends, family functioning), and focus more on coping strategies in the case of difficult parenting situations (e.g., bed time struggles).

In our study, parental expectations for children's sleep onset time were significantly associated with children's sleep quality parameters. Children of mothers with less adequate expectations for sleep onset time had longer settling period during which sleep rituals specific to the family may take place and spent more time in bed than they actually sleep. Although the children included in our study did not experience severe behavioral sleep problems, some children sometimes showed difficulties falling asleep or frequently woke up at night. In fact, children of mothers with less adequate expectations for children's sleep onset time had more frequently difficulties falling asleep than children of mothers with more adequate expectations. On one side it might be that differences between actual and expected sleep-wake patterns are themselves part of parent-reported behavioral sleep problems of the children (without a discrepancy parents would not usually indicate a problem), on the other hand they may contribute to children's sleep problems (which would have clear clinical implications). In the revised version of the International Classification of Sleep Disorders (ICSD-2 [23]) the rubric “dyssomnias, extrinsic sleep disorders” includes a sleep-onset association and a limit-setting sleep disorder. The latter involves bed time struggles and prolonged sleep onset time which may not be primarily driven by the caregivers' inability of setting limits, but rather by inadequate parental expectations. Although we cannot confirm this hypothesis with the present study, our clinical experience strongly supports this view.

The limitations of our study include the low participation rate which may be explained by the time-consuming procedure and the fact that our sample as a whole is only representative for white middle-upper class families (the low class was not represented). Furthermore, we note that our results are based on subjective reports which may be influenced by recall bias and are often vague, with statements such as “it depends on the situation” and with reporting to the nearest half hour or even full hour. Furthermore, using the mothers alone for all observations introduces potential rater bias. However, because health care professionals are usually confronted with subjective reports and high subjective parental distress may reflect the need for interventions, we focused on parents' perception and did not include objective sleep data such as actigraphy data.

To our knowledge this is the first study that describes parental expectations for children's sleep-wake patterns and explores their influence on children's sleep quality and mothers' distress. We conclude that the “goodness of fit” concept and parental expectations about their children's sleep play a key role in understanding normal and abnormal sleep during childhood.

## Figures and Tables

**Table 1:** Sample characteristics (*n* = 54).

Sex of children	
Male : female	56% : 44%
Birth order, mean (SD) [range]	1.6 (0.7) [1–3]
First born : second : third born	54% : 31% : 15%
Family size, mean (SD) [range]	2.3 (0.7) [1–4]
Only child : two children : >two children	7% : 67% : 26%
Socioeconomic status (SES)	
Middle class : upper class	48% : 52%
Temperament scores of children, mean (SD) [range]	
Emotionality	16.1 (3.8) [9–24]
Shyness	11.0 (4.7) [5–25]
Activity	19.3 (3.7) [9–25]
Sociability	19.5 (2.4) [12–24]
Parental distress scores, mean (SD) [range]	
Parental distress (PD)	23.2 (7.7) [13–48]
Parent-child dysfunctional interactions (P-CDI)	17.8 (3.9) [12–28]
Difficult child (DC)	25.9 (6.8) [13–46]
Total stress (TS)	66.9 (14.7) [39–105]

Percentages, mean (standard deviation) and [range] are reported.

**Table 2:** Descriptive statistics for parent-reported children's **actual ** and **expected ** sleep-wake patterns on scheduled (SC) and free days (FR; *n* = 54).

	*actual*		*expected*		Statistics^#^		
	SC*	FR*	SC	FR	Differences FR-SC	Differences *actual-expected *	Interaction
Bed time	20 : 05 (0 : 29)^ ×^	20 : 34 (0 : 47)	19 : 55 (0 : 25)	20 : 36 (0 : 46)	—	—	*P* = 0.000
Time of lights off	20 : 20 (0 : 34)^ ×^	20 : 47 (0 : 49)	20 : 05 (0 : 29)	20 : 44 (0 : 44)	—	—	*P* = 0.000
Sleep latency	0 : 14 (0 : 17)	0 : 12 (0 : 17)	0 : 12 (0 : 07)		NA	NA	NA
Sleep onset	20 : 34 (0 : 40)^ ×^	21 : 00 (0 : 53)	20 : 17 (0 : 31)	20 : 56 (0 : 47)	—	—	*P* = 0.000
Get-up time	7 : 17 (0 : 31)	7 : 55 (0 : 46)^ ‡^	7 : 16 (0 : 19)	8 : 13 (0 : 33)	—	—	*P* = 0.001
Sleep period	10 : 43 (0 : 37)	10 : 55 (0 : 41)^ ×^	10 : 59 (0 : 32)	11 : 17 (0 : 43)	0 : 14, *P* = 0.000	−0 : 20, *P* = 0.000	*P* > 0.05
Time in bed	11 : 13 (0 : 34)^ †^	11 : 20 (0 : 44)^ †^	11 : 21 (0 : 29)	11 : 37 (0 : 45)	0 : 13, *P* = 0.005	−0 : 11, *P* = 0.000	*P* > 0.05
Settling period	0 : 29 (0 : 27)^ ‡^	0 : 26 (0 : 28)	0 : 22 (0 : 18)	0 : 20 (0 : 16)	−0 : 03, *P* = 0.2	0 : 07, *P* = 0.001	*P* > 0.05
MSP	1 : 51 (0 : 32)^ †^	2 : 23 (0 : 46)	1 : 40 (0 : 28)	2 : 20 (0 : 43)			*P* = 0.027

Reported as mean (standard deviation), in hours : minutes.

*Wilcoxon-test between *actual* and *expected* sleep-wake patterns, separately for scheduled and free days.

^#^Twoway of analysis of variance for repeated measures with interaction. If the interaction was not significant, the results of the additive model are presented as differences. Otherwise the results are presented as averages and tested by the Wilcoxon-Test.

^×^ *P* ≤ 0.001, ^†^ *P* ≤ 0.01, ^ ‡^ *P* < 0.05.

NA: not applicable, SC: scheduled days, FR: free days.

**Table 3:** Actual sleep onset, actual get-up time, and parental expectation discrepancy (*Actual-Expected*) for these two sleep-wake patterns are presented by children's chronotype (CT score; *n* = 54).

		Morning Types (*n* = 18)	Neither Nor Types (*n* = 26)	Evening Types (*n* = 10)	Spearman Correlation
Sleep onset time (*actual*)	SC	20 : 10 (0 : 34)	20 : 37 (0 : 32)	21 : 10 (0 : 46)	*r* = 0.53, *P* = 0.000
	FR	20 : 23 (0 : 39)	21 : 10 (0 : 51)	21 : 38 (0 : 46)	*r* = 0.60, *P* = 0.000
Get-up time (*actual*)	SC	6 : 59 (0 : 23)	7 : 23 (0 : 32)	7 : 38 (0 : 25)	*r* = 0.54, *P* = 0.000
	FR	7 : 17 (0 : 27)	8 : 01 (0 : 40)	8 : 44 (0 : 29)	*r* = 0.74, *P* = 0.000
Sleep onset time (*actual-expected*)	SC	0 : 10 (0 : 13)	0 : 17 (0 : 23)	0 : 32 (0 : 43)	*r* = 0.31, *P* = 0.024
	FR	−0 : 08 (0 : 15)	0 : 08 (0 : 20)	0 : 23 (0 : 46)	*r* = 0.41, *P* = 0.003
Get up time (*actual-expected*)	SC	−0 : 08 (0 : 20)	0 : 06 (0 : 30)	0 : 07 (0 : 25)	*r* = 0.39, *P* = 0.004
	FR	−0 : 35 (0 : 32)	−0 : 17 (0 : 37)	0 : 08 (0 : 46)	*r* = 0.47, *P* = 0.000

Children's chronotype rated as “rather a morning type than an evening type”, “neither nor”, or “rather an evening type than a morning type” were summarized into the category “Neither Nor Types”.

SC: scheduled days, FR: free days.

**Table 4:** Children's sleep quality by parent report (*n* = 54).

Difficulties falling asleep	
No/rarely : sometimes : often	89% : 4% : 7%
Night waking	
No/rarely : sometimes : often	72% : 6% : 22%
Difficulties falling asleep and night waking	
No/rarely : sometimes : often	65% : 7% : 28%
Overall sleep quality, mean (SD) [range]	9.12 (1.32) [5–10], median = 10.0
Good : poor	96% : 4%

“No/rarely” in both behaviors, “often” in at least one behavior, “sometimes” describes the remaining situations.

**Table 5:** Associations between parental expectation discrepancy for children's sleep onset time and sleep quality parameters (*n* = 54).

	Correlations^#^	
	SC	FR
Settling period	*r* = 0.31^‡^	*r* = 0.47^×^
Time in bed without sleeping	*r* = 0.35^†^	*r* = 0.50^×^
Sleep latency	*r* = 0.23	*r* = 0.39^†^
Difficulties falling asleep	*r* = 0.33^†^	*r* = 0.21
Night waking	*r* = −0.01	*r* = 0.18
Overall sleep quality	*r* = −0.15	*r* = −0.23^‡^

^#^Spearman correlations were calculated for settling period, time in bed without sleeping, sleep latency, and overall sleep quality; Kendall correlations for difficulties falling asleep and night waking.

^×^ *P* ≤ 0.001, ^†^ *P* ≤ 0.01, ^‡^ *P* < 0.05.

SC: scheduled days, FR: free days.

**Table 6:** Linear regression analyses of parental distress (subscale P-CDI as dependent variable) on children's emotionality, chronotype, actual bedtime, time of lights off, sleep onset time, mothers' sleep quality, and parental expectation discrepancy for the 3 sleep-wake patterns on scheduled days (SC; *n* = 54).

	Statistics	
	*B*	*P* Value
Children's emotionality	0.43	*P* = 0.000
Children's chronotype	−0.12	*P* = 0.38
Children's bedtime (*actual*)	0.31	*P* = 0.04
Mothers' sleep quality	−0.23	*P* = 0.03
Parental expectation discrepancy for bedtime (*actual-expected*)	0.12	*P* = 0.37

Children's emotionality	0.43	*P* = 0.001
Children's chronotype	−0.12	*P* = 0.38
Children's time of lights off (*actual*)	0.30	*P* = 0.053
Mothers' sleep quality	−0.25	*P* = 0.04
Parental expectation discrepancy for time of lights off (*actual-expected*)	0.09	*P* = 0.52

Children's emotionality	0.438	*P* = 0.001
Children's chronotype	−0.115	*P* = 0.46
Children's sleep onset time (*actual*)	0.29	*P* = 0.108
Mothers' sleep quality	−0.26	*P* = 0.046
Parental expectation discrepancy for sleep onset time (*actual-expected*)	−0.003	*P* = 0.99

*B*: standardized coefficients.

Effects of sleep-wake patterns and of parental expectation discrepancy for children's sleep-wake patterns on scheduled days (SC) are presented.

## References

[1] Jenni OG, Werner H (2011). Cultural issues in children's sleep: a model for clinical practice. *Pediatric Clinics of North America*.

[2] Jenni OG, O’Connor BB (2005). Children’s sleep: an interplay between culture and biology. *Pediatrics*.

[3] Sadeh A, Anders T (1993). Infant sleep problems: origins, assessment, interventions. *Infant Mental Health Journal*.

[4] Thomas A, Chess S (1977). *Temperament and Development*.

[5] Thomas A, Chess S (1984). *Origins and Evolution of Behavior Disorders*.

[6] Owens J (2007). Classification and epidemiology of childhood sleep disorders. *Sleep Medicine Clinics*.

[7] Morrell J, Steele H (2003). The role of attachment security, temperament, maternal perception, and care-giving behavior in persistent infant sleeping problems. *Infant Mental Health Journal*.

[8] Sadeh A, Flint-Ofir E, Tirosh T, Tikotzky L (2007). Infant sleep and parental sleep-related cognitions. *Journal of Family Psychology*.

[9] Werner H, LeBourgeois MK, Geiger A, Jenni OG (2009). Assessment of chronotype in four- to eleven-year-old children: reliability and validity of the Children’s ChronoType Questionnaire (CCTQ). *Chronobiology International*.

[10] Roenneberg T, Kuehnle T, Pramstaller PP (2004). A marker for the end of adolescence. *Current Biology*.

[11] Hays RD, Kallich JD, Mapes DL, Coons SJ, Carter WB (1994). Development of the kidney disease quality of life (KDQOL(TM)) instrument. *Quality of Life Research*.

[12] Abidin R (1995). *Parenting Stress Index*.

[13] Tam K, Chan Y, Wong C (1994). Validation of the parenting stress index among Chinese mothers in Hong Kong. *Journal of Community Psychology*.

[14] Östberg M, Hagekull B (2000). A structural modeling approach to the understanding of parenting stress. *Journal of Clinical Child and Adolescent Psychology*.

[15] Buss A, Plomin R (1984). *Temperament: Early Developing Personality Traits*.

[16] Spinath F (2001). Temperamentsmerkmale bei Kindern. *Zeitschrift für Differentielle und Diagnostische Psychologie*.

[17] De Pauw SSW, Mervielde I, Van Leeuwen KG (2009). How are traits related to problem behavior in Preschoolers? Similarities and contrasts between temperament and personality. *Journal of Abnormal Child Psychology*.

[18] Largo RH, Pfister D, Molinari L, Kundu S, Lipp A, Duc G (1989). Significance of prenatal, perinatal and postnatal factors in the development of AGA preterm infants at five to seven years. *Developmental Medicine and Child Neurology*.

[19] Seitz J, Jenni OG, Molinari L, Caflisch J, Largo RH, Latal Hajnal B (2006). Correlations between motor performance and cognitive functions in children born < 1250 g at school age. *Neuropediatrics*.

[20] Landolt MA, Vollrath M, Ribi K (2002). Predictors of coping strategy selection in paediatric patients. *Acta Paediatrica, International Journal of Paediatrics*.

[21] Paykel ES, Emms EM, Fletcher J, Rassaby ES (1980). Life events and social support in puerperal depression. *British Journal of Psychiatry*.

[22] Steinhausen H, Winkler Metzke C (2001). The Zurich life event list (ZLEL): findings from an epidemiological study. *Kindheit und Entwicklung*.

[23] Medicine, A.A.o.S., The International Classification of Sleep Disorders

